# Tick-borne encephalitis in Japan, Republic of Korea and China

**DOI:** 10.1038/emi.2017.69

**Published:** 2017-09-20

**Authors:** Kentaro Yoshii, Joon Young Song, Seong-Beom Park, Junfeng Yang, Heinz-Josef Schmitt

**Affiliations:** 1 Laboratory of Public Health, Graduate School of Veterinary Medicine, Hokkaido University, Sapporo 060-0818, Japan; 2 Division of Infectious Diseases, Department of Internal Medicine, Korea University Guro Hospital, Gurodongro 148, Gurogu, Seoul 08308, Republic of Korea; 3 Pfizer Pharmaceuticals Korea Ltd, Seoul 100-771, Republic of Korea; 4 Pfizer Investment Co., Ltd. The Fifth Square, Tower B, 9/F, No. 3-7, Chaoyangmen North Avenue, Dongcheng District, Beijing 100010, China; 5 Scientific Affairs, Pfizer Vaccines Europe, 23-25 Avenue du Dr Lannelongue, Paris 75014, France

**Keywords:** East Asia, tick-borne encephalitis, tick-borne encephalitis virus

## Abstract

Tick-borne encephalitis virus (TBEV) causes mild or moderate febrile illness in humans that may progress to encephalitis, leading to severe long-term complications and sometimes death. TBEV is prevalent in the Eurasian continent and has been isolated in China, Japan and Republic of Korea (ROK). The TBEV isolates from Japan are of the Far-Eastern subtype; in ROK, the isolates are of the Western subtype; and all TBEV isolates in China are of the Far-Eastern subtype, except one strain that was identified most recently as the Siberian subtype. TBE is endemic to the northeast, northwest and southeast of China; only two confirmed TBE cases have been reported in Japan to date; and no TBE case has been confirmed in ROK. For TBE patients in China, the onset of disease is acute with no biphasic course for disease presentation. The clinical spectrum of disease phenotypes may be wider than currently understood, since serological evidence suggests the presence of TBEV infections in healthy people, indicating that asymptomatic or unspecific manifestations of TBEV infection may exist. The current treatment for TBE is supportive care. In China, vaccines against TBEV have been developed and are available with demonstrated immunogenicity and safety, although efficacy data are lacking. No vaccines are available in ROK or Japan.

## INTRODUCTION

Tick-borne encephalitis (TBE) usually presents as a moderate febrile illness that is followed by encephalitis in ~40% of patients. TBE was first reported in Austria and Russia in the 1930s.^1^ TBE is caused by TBE virus (TBEV), a single-stranded, positive-sense RNA virus of the genus *Flavivirus*, and is prevalent in the Eurasian continent, including Central Europe, Russia, Kazakhstan, Kyrgyzstan, Mongolia and Far-Eastern Asia, China, Korea and Japan.^1, 2, 3^ TBEV has been divided into three subtypes based on phylogenetic analysis: the Far-Eastern subtype, formerly Russian spring-summer encephalitis virus; the Western or European subtype, formerly the Central European encephalitis virus; and the Siberian subtype (known as West Siberian virus).^1, 3^ The primary vector of the Western subtype is *Ixodes ricinus*, while *I. persulcatus* is the primary vector of the Far-Eastern and Siberian subtypes.^3^ In nature, TBEV is maintained between ticks and wild vertebrate hosts; small mammals, such as rodents and hedgehogs, are its main reservoirs.^3^ Larger animals, such as deer, horses or livestock, and birds, are not considered to play an important role in the natural transmission of TBEV between ticks.^3^ However, since TBEV has been detected in ticks carried by migrating birds,^4^ it has been suggested that migrating birds contribute to the geographic spread of TBEV-infected ticks. Humans are not involved in the natural transmission of TBEV and are only accidental hosts.

In East Asia, TBE patients were first reported in China in 1943, and TBEV was isolated one year later from brain samples of patients, as well as from *I. persulcatu*s.^5, 6^ In the summer of 1952, a TBE outbreak was recorded among forest workers in northeast China with a mortality rate of over 20%. TBEV was isolated from the brain tissues of the deceased patients. Further epidemiological surveys in the endemic forest areas found TBEV-infected rodents, and TBEV was isolated from local tick species, including *I. persulcatus*, *Haemaphysalis concinna*, *Dermacentor silvarum* and *H. japonica*.^6^ Since then, TBE has been recognized in China, and endemic areas have been identified in the northeast, northwest and southwest of China. Meanwhile, vaccines against TBEV have been developed and used in China since the early 1950s—soon after the virus was first identified, and different vaccine types have been employed in different periods.

In Japan, patients infected with a virus within the TBE serocomplex had encephalitis during an epidemic of Japanese encephalitis (JE) in 1948 in the Tokyo area. The virus that was isolated was named Negishi virus, which was retrospectively identified as a member of the louping ill virus through antigenic and phylogenetic analysis conducted decades later.^7, 8^ No TBE case had been reported in Japan since then until 1993, when a case of viral encephalitis in Hokuto City in southern Hokkaido was diagnosed as TBE.^9^ Subsequently, TBEV was isolated from dogs and *I. ovatus* in the area where the patient lived, and the virus strains were identified as the Far-Eastern subtype.^10, 11^ It was only in July 2016 when the second case^12^ and in 2017 the third and the fourth cases of TBE were confirmed in Hokkaido. No other TBE cases have been reported in Japan since 1993, although endemic foci of TBEV were detected, particularly in southern Hokkaido.

Anecdotal information and locally published literature suggest that TBEV and TBE may be more widespread than understood and, in particular, that the virus may exist in more southern locations in Asia.^13^ Over the last decade, TBEV has been isolated in the Republic of Korea (ROK) from ticks, including *H. longicornis*, *H. flava* and *I. nipponensis*.^14, 15, 16, 17, 18^ In addition, TBEV has been isolated from *Apodemu agrarius* and wild boars.^14^ Contrary to those from neighboring countries, that is, Japan, China and northeastern Russia, in which only the Far-Eastern subtype TBEV was isolated,^2, 10, 19, 20^ the TBEV isolates from ROK were identified, as the Western subtype by DNA sequence and phylogenetic analyses.^15, 16, 17^ No human TBE cases have been reported to date in ROK.

**Figure 1:**
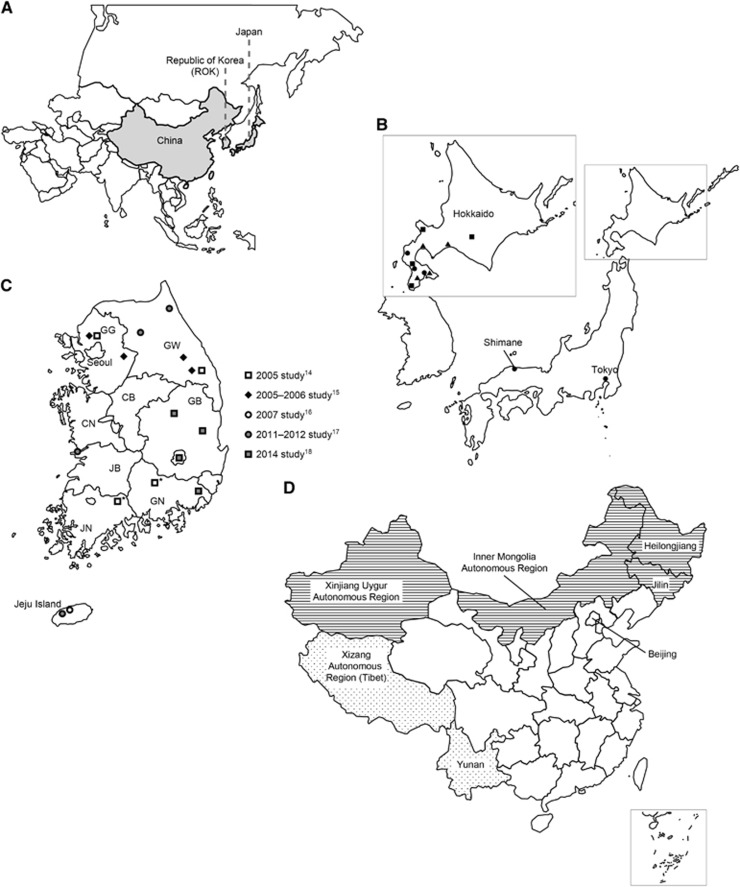
Epidemic regions of tick-borne encephalitis virus and/or tick-borne encephalitis in Far-Eastern Asia, China, the Republic of Korea and Japan. (**A**) Map of Asia. Countries of interest, that is, China, Republic of Korea and Japan, are shaded in gray. (**B**) Geographic locations of epizootiological survey sites, showing tick-borne encephalitis virus prevalence in Japan. Closed circles, triangles and squares represent the points where seropositive rodents, dogs and horses for tick-borne encephalitis virus were detected, respectively. (**C**) Locations in the Republic of Korea where tick-borne encephalitis virus (TBEV)-positive ticks or wild rodents were identified. Symbols on the map indicate regions where TBEV-positive ticks were identified from individual studies (with reference numbers). Asterisks indicate regions where TBEV-positive rodents were identified. CB, Chungcheongbuk-do; CN, Chungcheongnam-do; GB, Gyeongsangbuk-do; GG, Gyeonggi-do; GN, Gyeongsangnam-do; GW, Gangwon-do; JB, Jeonllabuk-do; JN, Jeonllanam-do. (**D**) Geographic distribution of tick-borne encephalitis in China. Areas with horizontal lines represent the provinces or autonomous regions where tick-borne encephalitis (TBE) virus was isolated and human TBE cases were reported. Dotted regions indicate provinces or autonomous regions where TBE is suspected based on serology data.

To fully understand the potential burden of TBE infection in East Asian countries, including China, Japan and ROK (Figure 1A), and to guide further epidemiological research, as well as the development of an effective control strategy for TBE in this region, the literature was reviewed to obtain systematic information regarding TBEV and TBE in these three countries. This information included TBEV isolation and characterization, TBE clinical presentation and treatment, epizootiological survey and sero-surveillance programs and vaccine development and application.

## TBE IN JAPAN

### First confirmed case of TBEV infection in Japan

In Japan, the Japanese encephalitis virus (JEV), a mosquito-borne flavivirus, has been widely endemic, and new JE patients have been reported annually. A virus was isolated from a patient with encephalitis during the JE endemic in 1948 in the Tokyo area and named Negishi virus; this virus was first identified as a member of the Russian spring-summer encephalitis virus family^7^ and was later found to be more closely related to the louping ill virus than to other tick-borne viruses through antigenic and phylogenetic analysis.^8^ However, no TBE cases had been reported until 1993, and it was believed that in Japan there was no endemic focus of tick-borne flaviviruses in the absence of further epidemiological studies.

In 1993, a case of viral encephalitis in Hokuto City, in the southern part of Hokkaido, was eventually diagnosed as TBE. The patient had a fever, headache and neurological symptoms, such as seizures. A hemagglutination inhibition (HI) test against JEV showed significantly increased HI antibodies. However, 2-mercaptoethanol-sensitive HI antibodies were not detected, and it was unlikely that JEV infection occurred in Hokkaido, where JEV was not endemic. Furthermore, blood feeding by vector mosquitoes was unlikely because they were not active at the end of autumn in that area. Further serological analysis was conducted to examine antibodies against other flaviviruses. In enzyme-linked immunosorbent assay (ELISA) testing for IgM antibodies and neutralization tests, the antibody titer against JEV was very low, while high titers of antibodies against TBEV were detected. These results indicated that the patient was infected with TBEV.^9^

### Isolation of TBEV in Hokkaido

Because the aforementioned patient was a dairy farmer and had no history of overseas travel, it was reasoned that she was infected with TBEV through a tick bite in her area of residence in Hokkaido. In 1994, a serological survey was conducted on cattle and dogs in areas surrounding the patient’s farm. No antibodies against TBEV were detected in the serum samples from cows, suggesting that the infection route was not via raw milk from TBEV-infected cows. Eight of the nine dogs from the surrounding farms showed high titers of neutralizing antibodies (>640) against TBEV. In 1995, five of ten sentinel dogs kept locally in a free-living environment had seroconverted between April and May, and TBEV was isolated from the blood of three seroconverted dogs. The isolated viruses were recognized by antibodies specific to the TBE-serocomplex viruses. Sequence and phylogenetic analysis revealed that the viruses were of the Far-Eastern subtype of TBEV.^10, 21^ Later, TBEV was also isolated from ticks and rodents in Oshima, located in southern Hokkaido.^22^

### Vectors of TBEV in Hokkaido

Hokkaido is the northernmost island of Japan; it is close to the Far-Eastern part of Russia, where the Far-Eastern subtype of TBEV is endemic. Biogeographical and ecological conditions are similar in both areas, including vector ticks and the TBEV host reservoir. In the northern part of Hokkaido, *I. persulcatus* is predominant and is the main tick vector of the Far-Eastern subtype of TBEV, whereas *I. ovatus* is predominant in the southern part of Hokkaido (Table 1).^29^

To determine the principal tick vector of TBEV in the residence area of the patient, a survey of TBEV in ticks was conducted. *I. ovatus* was found to be the most common tick species. TBEV was isolated from *I. ovatus*, and the minimum field infection rate was calculated as 0.33%.^11^ These results indicated that *I. ovatus* was the most likely vector species of TBEV in this area. However, it has also been reported that other predominant ticks might serve as vectors.^30^

### Epizootiological survey of TBEV in Japan

A wide range of animal species are infected with TBEV through bites from TBEV-infected ticks. To determine the prevalence of TBEV in Japan, serological surveys of mammals were conducted. In Hokuto City, where the first TBE patient was detected, anti-TBEV antibodies were also detected in *A. speciosus*, *A. argenteus*, *Myodes rufocanus* and *Rattus norvegicus* in 1995, 1996 and 2008, indicating that the endemic foci of TBEV has been maintained over a long period.^31, 32^ Furthermore, anti-TBEV antibodies were detected in small mammals, horses and dogs in various parts of central to southern Hokkaido, where *I. ovatus* ticks are predominant (Figure 1B), indicating that TBE foci are distributed over a wide area of Hokkaido.

Wild vertebrates that are potential hosts for TBEV and *I. ovatus* ticks are widely distributed in various regions of Japan. Large-scale epizootiological surveys were conducted in wild animals from various areas, including the three major islands Hokkaido, Honshu and Shikoku, to determine the endemic areas of TBEV. Rodents seropositive for anti-TBEV antibodies were detected in Shimane Prefecture in the western part of Honshu Island.^32^ Other wild animals with antibodies against TBE-serocomplex viruses have also been detected in the western parts of Honshu Island (Figure 1B).

The analysis of the viral envelope protein genes using average substitution rate and synonymous distances suggested that TBEV strains in Japan diverged from ancestor strains in Far-Eastern Russia ~260–430 years ago.^22^ Nevertheless, it is not clear how TBEV was introduced into Japan. One possibility is that migratory birds might have transported TBEV-infected ticks from Far-Eastern Russia to Japan, since it has been reported that TBEV-infected ticks were detected on migratory birds^4^ and that *Ixodes* ticks were detected on several migratory bird species along the migratory route in Japan.^33^ A second possibility is that a small reservoir of mammals infected with TBEV might have migrated from the Eurasian continent to Japan. Considering that the Japanese islands were once connected to the Asian continent and were separated at least 10 000 years ago, the reservoir animals might have been transported through human transportation vehicles.

### Virulence of TBEV isolated in Japan

The Japanese TBEV isolates, the Oshima strains, showed a nucleotide difference of ~4%–6% from strains isolated in Far-Eastern Russia.^21, 22, 34^ Compared with a prototype strain of the Far-Eastern subtype, Sofjin-HO, the Oshima strain (Oshima 5–10) replicated more slowly and was less virulent in conventional laboratory mice.^35^ Most inbred laboratory mice do not possess a functional 2'–5'-oligoadenylate synthetase 1b (*Oas1b*) gene, which was previously identified as a flavivirus resistance gene. In a congenic strain carrying a functional *Oas1b* gene, the intracerebral infection of the Oshima strain (Oshima 5–10) resulted in limited signs of illness, while infection of Sofjin-HO resulted in death with severe neurologic signs. These findings indicated that the Oshima strain was more susceptible to the antiviral activity of Oas1b against neurovirulence.^36^

Analysis of the virulence factors of TBEV using reverse genetics systems revealed that amino acid differences in non-structural proteins, but not in viral envelope proteins, were involved in the attenuation of the Oshima strain (Oshima 5–10).^37^ Furthermore, a deletion in the variable region of the 3' untranslated region increased the virulence of the Oshima strain.^37^ Most of the TBEV strains isolated from ticks and wild rodents contained no deletions in the variable region, but deletions in the variable region were found in strains passaged in mammalian cell culture^38^ and in strains isolated from human patients.^39^ This suggested that the deletions are caused by viral adaptation to mammalian cells and are related to virulence in severe cases in humans. Therefore, the possibility cannot be dismissed that the Japanese TBEV might obtain increased virulence in humans by adaptation during infection in humans.

### Problems controlling TBEV infection in Japan

In the Prevention of Infectious Diseases and Medical Care for Infectious Patients Act that became legislation in Japan in 1999, TBE was classified as a reportable infectious disease and TBEV was classified as a Class 3 select agent handled under bio-safety level (BSL) 3 conditions. Nevertheless, only three TBE cases (one in 2016, two in 2017) have been reported since the first confirmed TBE cases in 1993, although endemic foci of TBEV have been detected in various parts of Japan, particularly in the southern parts of Hokkaido.

This low occurrence of TBE in Japan might be attributed in principle to the following three factors: (1) The possibility of receiving a tick bite has been reduced by the progress of urbanization, and no-one has actually been infected with TBEV, although it is arguable that endemic foci of TBEV have been located in areas that people, particularly farmers and forest workers, access easily, even on a daily basis. There is an urgent need to conduct serological surveys among residents in TBEV-endemic areas in Japan to establish adequate preventive measures. (2) Japanese TBEV may be of low virulence against humans, and TBEV infection might be asymptomatic or cause only febrile illness without encephalitis symptoms. This speculation has been supported by the fact that the Japanese TBEV isolates in Hokkaido demonstrated lower virulence in mice, as previously described, than other TBEV strains of the Far-Eastern subtype. (3) TBE patients might not be recognized and diagnosed correctly. One major reason for this is the low awareness of TBE in Japan, even among medical doctors (Dr Yoshii, personal communication). Other reasons include the difficulty in conducting laboratory examinations for the diagnosis of TBEV infection, as certain examinations are only available in limited facilities because of the low awareness of the disease and the restrictions in handling TBEV as a BSL 3 agent. However, in recent studies, diagnostic methods that can be conducted in BSL 2 facilities have been developed in Japan using subviral particles as diagnostic antigens.^40, 41^ Considering that BSL 2 facilities are more common than BSL 3 facilities, these methods will increase the number of laboratories that can perform examinations of TBEV and will therefore help to improve awareness of TBE.

To control TBEV infection, it is important to identify TBEV-endemic areas and to design an effective prevention plan, including vaccination. Formalin-inactivated vaccines based on the European subtype have been used in European countries and have been shown to induce highly protective immunity. In a mouse model, one of these vaccines was shown to prevent infection with the Japanese TBEV isolates of the Far-Eastern subtype.^42^ Currently, none of these vaccines is licensed in Japan.

## TBE IN ROK

### TBEV isolated in ROK

In ROK, human encephalitis cases with unknown causes have been increasingly reported, although no cases of TBE have been reported. Over the last decade, various studies have been carried out to determine whether TBEV is present in ROK. In 2008, TBEV was first reported to have been isolated from wild rodents (*A. agrarius*) captured in Hapcheon, Gyeongsangnam-do, by virus isolation using suckling mice. These TBEV isolates (KrM216, KrM219) caused symptoms of encephalitis in suckling mice and were able to grow from brain preparations in cell culture.^14^ In addition, the envelope E gene of TBEV from ticks (*H. longicornis*: *n*=548; *I. nipponensis*: *n*=87) and mammalian hosts (*A. agrarius*: *n*=24; wild boars: *n*=16) was amplified using reverse transcriptase-nested polymerase chain reaction (RT-nested PCR). *H. longicornis* was collected from Dongducheon, Geyonggi-do and Jeongseon, Gangwon-do; *I. nipponensis* from Jeongseon, Gangwon-do; and *A. agrarius* from Hapcheon, Gyeongsangnam-do and Gurye, Jeollabuk-do.^14^ Unexpectedly, phylogenetic analysis revealed that the TBEV isolates belonged to the Western subtype of TBEV, while in the neighboring countries, including Japan, China and northeastern Russia, only the Far-Eastern subtype was isolated.^14^

### Geographical distribution of TBEV and vector ticks in ROK

Ticks have been shown to transmit various zoonotic pathogens in ROK, such as *Anaplasma* spp.,^43, 44^ *Bartonella* spp.,^44^ *Borrelia* spp.,^44^ *Ehrlichia* spp.,^43^ *Rickettsia* spp.,^43^ severe fever with thrombocytopenia syndrome virus (SFTSV),^18^ and TBEV.^14, 15, 16, 17^ In the wild in ROK, *Haemaphysalis* spp. were found to be the predominant species using dragging techniques, while *I. nipponensis* became predominant when harvested from small mammals.^16, 45^

To date, four surveillance studies have been conducted to evaluate the prevalence of TBEV in ticks in ROK. The first study was conducted in 2005–2006 in 12 regions of five provinces of ROK (Figure 1C).^15^ A total of 2460 ticks were collected from grassland and forest by dragging and flagging (*n*=1878) or were removed from wild and domestic animals (*n*=582). RT-nested PCR targeting the E protein gene of TBEV showed a positive result in 10 adult ticks (0.4%, 10/2104) and 2 nymph ticks (0.2%, 2/356) collected from four regions in two provinces (Table 1). Phylogenetic analysis of sequences obtained clustered the E gene of these TBEV within the Western subtype with 98% identity.^15^ Later, this research team, whose work was previously described, also obtained the first TBEV strains in ROK that could cause encephalitis in mice.^14^

The second investigation was conducted in 2007 to evaluate the potential occurrence of TBEV infection in the southern provinces of ROK, including Jeju Special Self-Governing Province (Jeju Island), Jeollanam-do, Gyeongsangbuk-do and Gyeongsangnam-do (Figure 1C).^16^ Ticks were collected using dragging techniques at 113 sites representing different habitats, including forest left-litter, herbaceous vegetation, and grasses. Among the 6788 ticks collected, 4077 were pooled (649 pools) by collection site, species and developmental stage and subjected to RT-nested PCR to detect the E gene of TBEV. Positive results were obtained in six pools of nymphs of *H. longicornis* and *H. flava* collected from Jeju Island (Table 1). For the two most common tick species, *H. longicornis* (73.4%, 4984/6788) and *H. flava* (22.4%, 1523/6788), the minimum field detection rates (assuming at least one TBEV infected tick per pool) were 0.17% and 0.14%, respectively. For the other tick species harvested, *H. phasiana*, *Amblyomma testudinarium*, *I. nipponensis* and *I. turdus*, regardless of developmental stage or collection site, no TBEV E gene was detected. Similarly to the previous study, phylogenetic analysis based on the NS5 and E genes identified the Jeju strains as the Western subtype of TBEV.^16^

A larger-scale surveillance study was then conducted during 2011–2012 in 25 localities of 10 provinces of ROK (Figure 1C). For this study, ticks were collected from various vegetative habitats, including grass thicket, grassland and broad-leaved and coniferous forests, using flagging and dragging or BG-Sentinel Trap.^17^ There were 13 053 ticks collected with *H. longicornis* (90.8%, 11 856/13 053) as the most abundant species; the rest included *H. flava* (8.8%, 1149/13 053), *I. niponensis* (0.3%, 42/13 053), and *I. persulcatus* (0.05%, 6/13 053). On the basis of collection site, species and developmental stage, the ticks were grouped into 1292 pools, and the presence of the E gene of TBEV was examined using RT-nested PCR. Ten pools (7 *H. longicornis*, 2 *H. flava* and 1 *I. niponensis*) produced positive PCR products (Table 1, Figure 1C). Assuming that a positive pool contains a minimum of one TBEV-infected tick, the minimum infection rates for *H. longicornis*, *H. flava*, and *I. nipponensis* were 0.06%, 0.17% and 2.38%, respectively. Unsurprisingly, compared with known TBEV strains in phylogenetic analysis, the sequences obtained from TBEV-positive products were identified as the Western subtype.^17^

More recently, a fourth surveillance study on ticks was carried out in 2014 with the aim of understanding the prevalence of TBEV and other tick-transmitted viruses, including Powassan virus (POWV), Omsk hemorrhagic fever virus (OHFV), Langat virus (LGTV) and severe fever with thrombocytopenia virus (SFTSV).^18^ There were 21 158 ticks collected from vegetation by dragging at 139 sites in 6 provinces (Gyeonggi-do, Gangwon-do, Chungcheongnam-do, Gyeongsangbuk-do, Gyeongsangnam-do and Jeju Island) and 4 metropolitan areas (Seoul, Daegu, Ulsan and Busan) between March and October 2014. *H. longicornis* (83.04%, 17 570/21 158) was the dominant tick species, while other tick species, *H. flava* (15.68%, 3317), *I. nipponensis* (1.18%, 249), *Am. testudinarium* (0.05%, 11) and *H. phasiana* (0.04%, 8), were much less common. Although SFTSV RNA was detected in *H. longicornis*, *H. flava* and *I. nipponensis*, the viral RNA for POWV, OHFV, and LGTV was not detected. In addition, TBEV was detected by RT-nested PCR in *I. nipponensis, H. flava* and *H. longicornis* collected from grass and pine forest habitats or grass habitats in 4 regions (Table 1, Figure 1C). As in the previous studies, phylogenetic analysis confirmed that the TBEV strains identified in this study belonged to the Western subtype.^18^

### Current understanding of TBEV prevalence in ROK

Over the last several years, although no human TBE case has yet been reported in ROK, epidemiological and virological characteristics of TBEV have been investigated. Ixodid ticks have been identified as the main vectors for TBEV;^14, 15, 16, 17, 18^ TBEV strains that could cause encephalitis in suckling mice have been isolated from wild rodents;^14^ and based on the E gene, all of the TBEV strains reported were clustered with the Western subtype using phylogenetic analysis.^14, 15, 16, 17, 18^ On the basis of the molecular evidence, it is understood that TBEV strains endemic to ROK are different from those identified in neighboring countries such as China, Japan, and northeastern Russia, since those strains all belong to the Far-Eastern subtype.^19, 20, 22^ Geographically, certain regions in ROK have been identified as potential endemic areas for TBEV infection, including Dongducheon, Jeongseon, Hapcheon, Gurye, Gyeonggi-do, Gangwon-do, Jeollabuk-do and Jeju Island (Figure 1C).^14, 15, 16, 17, 18^

Overall, although there has been no confirmed human case of TBEV infection in ROK, TBEV can be endemic to various localities, and *H. longicornis*, *H. flava* and *I. nipponensis* may be potential vectors of the Western subtype of TBEV found in these areas.

### TBEV prevention in ROK—Limitations and challenges

The main limitation of TBEV surveillance in ROK is the low awareness of the disease, with limited diagnostic practice in clinical settings. The incubation period of TBE ranges from 4 to 28 days, with an average of 7–10 days. The disease is typically biphasic in most patients infected with the Western subtype of TBEV (all the TBEV isolates from ROK belong to this subtype). The first stage usually presents muscle pain, fatigue, headache and a fever lasting 2–7 days, which peaks at a temperature of 37.5–39.0 °C. There are no signs or symptoms of meningoencephalitis during this phase.^45^ To diagnose TBEV infection, either immunofluorescence antibody assay (IFA) or ELISA is required for anti-TBEV antibody screening, followed by a TBEV-specific plaque reduction neutralization test (PRNT) to confirm the positive results with the detection of neutralization antibody. Not all clinicians in ROK are aware of the symptoms of TBE, and the specific laboratory tests required are not widely available.

Korean Centers for Disease Control and Prevention has been screening most encephalitis cases with unknown causes for certain rare diseases, including West Nile virus infection, Dengue fever, chikungunya and yellow fever; since 2006, TBE has also been evaluated. The Neurovirus Laboratory in the National Institute of Health of ROK conducts such TBEV screening on clinical samples (for example, blood and cerebrospinal fluid) sent by hospitals using IFA or ELISA followed by PRNT or PCR for confirmation. As of 2016, no TBE cases had been detected. However, because of the short duration of TBE viremia in the infected host, it is not easy to confirm infection using blood and CSF samples collected at later clinical stages. In addition, false-negative TBE results are possible among unidentified clinical cases.

Within several years, evidence of tick species being potential carriers of TBEV has emerged throughout ROK, and the molecular characteristics of prevalent TBEV have been defined. These results indicate that the potential burden of TBEV in ROK is continually increasing. However, it is most likely that the endemic regions of TBEV are being under estimated because of the relatively low awareness of the disease and the difficulties associated with the diagnostic process. A similar situation has been observed for another tick-borne disease, severe fever with thrombocytopenia syndrome (SFTS), which is an emerging infectious disease caused by SFTSV that has been detected in northeast and central China.^46^ Since the first publication on SFTS and SFTSV in 2011,^46^ which provided a detailed description of the clinical symptoms of SFTS and the isolation and molecular characterization of the causal virus, the awareness of this disease has increased. As a result, increasing numbers of SFTS cases have been reported, and SFTSV has been isolated in various localities in Japan and ROK.^18, 47^ This experience with SFTS demonstrates that the severity of an endemic infectious disease can only be revealed when full awareness of the disease is achieved.

Therefore, to fully understand the threat posed by TBE and TBEV to public health, as well as to domestic animals and wildlife, further research on the disease, the causal virus, and the vector is needed. Investigations should emphasize generating a clear epidemiologic profile of TBE in a given country, characterizing TBEV isolated from infected ticks and evaluating its pathogenesis in hosts, and obtaining serologic evidence of TBE prevalence in high-risk populations, such as farmers in the endemic area or encephalitis patients negative for JEV. Findings from such studies will help to reduce the number of false-negative cases in clinical settings and to increase the probability of making a correct diagnosis in asymptomatic TBEV-infected cases from potential endemic regions.

## TBE IN CHINA

### TBE epidemiology in China

TBE, also known as Forest Encephalitis in China, is endemic to northeast China and is also present in northwest and southwest China (Figure 1D). In northeast China, confirmed TBE patients (that is, with TBEV isolated from tissues obtained from the patients) have been reported from two provinces, Heilongjiang and Jilin, where TBEV has also been isolated from *M. rufocanus*, *A. sylvaticus* and *Eutamias sibiricus*.^5, 6^ A third TBE endemic region identified is the Inner Mongolia Autonomous Region.^23^ In the northwest, TBE patients have been confirmed in the Xinjiang Uygur Autonomous Region.^24, 25^ In addition, TBE cases have been reported in Yunnan Province.^26^ Overall, the forest areas in the northeast are considered the most important TBE-endemic areas in China because TBEV has been frequently isolated in these areas.^6^

Natural foci of TBE have been found in the northeast (Changbaishan, Daxing'anling and Xiaoxing'anling), the northwest (Tianshan and Altay Mountains) and the southwest (forest areas of the Hengduan Mountain Range and Gaoligongshan).^23, 26^ In the northern foci, *I. persulcatus* is the dominant tick species and the main vector of TBE, although other tick species, including *H. concinna*, *H. japonica* and* D. silvarum*, have also been identified as vectors for TBE.^5, 6^ However, in the southwest natural foci in Yunnan Province, *I. persulcatus* has not been found, but *I. ovatus* has been identified as the dominant vector for TBE.^26^ Among vector ticks collected from different endemic regions, TBEV infection rates vary substantially. For instance, one study from Xinjiang (northwest China) reported that 14.3%–47.7% of the *I. persulcatus* examined carried TBEV,^24^ while another study from Heilongjiang (northeast China) found that ~4.45% of the *I. persulcatus* were TBEV-positive.^27^ Moreover, even within the same endemic region, free-living ticks collected from two similar habitats in a conifer broad leaf forest area 2 km apart showed a significant difference in TBEV infection rate (4/21 and 0/20).^5^ A trend in which the endemic foci of TBE spread from forestry areas toward agricultural areas has been observed.^48^ Alarmingly, following climate warming, urbanization, strategy changes in land use, and increased human outdoor activities, the geographical distribution of *I. persulcatus* has been found to have spread to more provinces and municipalities in China, including the Ningxia Hui Autonomous Region, Hebei, Tianjin, Beijing and Henan.^49^

Until very recently, phylogenetic analyses based on the E gene of TBEV demonstrated that all TBEV strains isolated in China belonged to the Far-Eastern subtype.^20, 28^ In 2016, the Siberian subtype of TBEV was first detected in China using Illumina deep sequencing-based screening from samples of *I. persulcatus* collected in the Xiaerxili Natural Reserve, a TBE natural focus in the Xinjiang Uygur Autonomous Region (northwest China).^50^ Viral RNA was amplified using RT-PCR, and phylogenetic analysis confirmed that the similarities between the TBEV isolate and the Siberian, the Far-Eastern and the Western subtype were 95.5%, 87.8% and 87.4%, respectively.^50^ Considering that global climate change may cause TBE foci to spread from higher latitudes towards lower latitudes, there is a risk of importing the Siberian subtype of TBEV in the north (Inner Mongolia) and the northwest (Xinjiang) of China. However, because the Far-Eastern subtype of TBEV has been previously isolated in Xinjiang,^28^ this latest identification of the Siberian subtype of TBEV indicates that co-circulation of the two subtypes may have already started in China. Co-circulation of all three TBEV subtypes has been observed in Latvia.^51^

TBE is a notifiable disease in Heilongjiang Province, where records show an incidence rate of 3.17 per 100 000 in 1952 and 0.01–1.04 per 100 000 from 1953 to 1998; in more recent years (from 1980 to 1998), the average incidence was 0.33 per 100 000.^52^ In the Daxing'anling endemic foci in the Inner Mongolia Autonomous Region, the incidence of TBE was 88.6 per 100 000 from 1990 to 1998.^53^ TBE cases mainly occurred in spring and summer, 2–4 weeks after the vector ticks become active,^23, 53^ although TBE cases have been reported in winter.^52^ On the basis of limited data, a secular trend in TBE prevalence was speculated, as it appeared that a peak in TBE incidence occurred every 7–8 years between 1952 and 1998.^52^

In China, TBE has been considered an occupational disease, as most patients (~70%) have been forest workers.^52^ However, since the 1980s, particularly in the late 1990s, the occupations of TBE patients changed considerably; 70%–95% of the patients were non-forest-working farmers, housewives, domestic workers, students or anyone with any occupation who entered the endemic forest areas.^13, 52^ Patients with TBE may have entered tick habitats because of a continual increase in leisure activities (such as walking and rambling, trekking).

Serological surveys have been carried out in TBE-endemic areas in China, providing evidence of the existence of asymptomatic TBEV infections. In general, anti-TBEV antibodies were detectable in 0.99% to 19.7% of healthy adults.^27^ For those who had a history of tick bites but did not show any symptoms of TBE, the rate of IgG-positivity varied from 1.1% in the Daxing'anling area in Inner Mongolia^54^ to 35.4% (23/65) among the nomads and staff of the nature reserve in the Xiaerxili Natural Reserve in Xinjiang.^25^ Among patients with fever in the Gaoligongshan region in Yunnan, 30.55% had anti-TBEV antibodies.^26^ In addition to humans, anti-TBEV antibodies have also been detected among domestic animals and livestock, as well as wild mammals. In the northeast and northwest TBEV-endemic areas, small rodents, such as *Dryomys nitedula*; medium-sized mammals, such as *Marmota baibacina*; and larger mammals, such as *Capreolus capreolus*, *Vulpes vulpes*, and *Sus scrofa*, have been found to have anti-TBEV antibodies.^5, 24^ In Yunnan Province (southwest China), apart from small rodents, anti-TBEV antibodies have also been found in *Macaca mulatta* and *Rousettus leschenaultia*.^26, 55^

Furthermore, in the Xizang (Tibet) Autonomous Region, serology studies have indicated TBEV infections in the Linzhi and Milin areas,^20, 27^ although TBEV has not yet been isolated.

### Disease presentation of TBE in China

In China, TBE is diagnosed based on the criteria published by the Ministry of Health,^56^ including (1) a history of being in forest areas in spring and summer with a history of tick bite(s), (2) sudden fever, (3) symptoms indicating acute central nervous system impairment, (4) positive results on TBEV-specific serological tests (IgM positivity or a fourfold rise in IgG in the convalescent period compared with that in the acute period), (5) epidemiological evidence indicating endemic TBE in the areas where the patient worked or visited and (6) the exclusion of other causes such as JEV, poliomyelitis or other viral encephalitis.

TBE patients in China generally had a sudden high fever (39–49 °C) that lasted for 5–7 days (or up to 12–14 days), ~1 week (incubation period 4–28 days with 8 days as the average) after having tick bites. Patients usually had nausea, vomiting, severe headache and neck stiffness, while some had paralysis.^27^ An analysis that included 380 TBE patients found that two-thirds (257/380) were severe cases with the presence of muscle paralysis, disturbance of consciousness and difficulties in swallowing and verbal communication.^57^ In addition, impairment of the eye muscles,^58^ myocardial damage^59^ and liver function impairment^60^ were present in TBE patients. However, for patients with the mild form of TBE, no signs of nervous system damage were observed. Moreover, no indication of a biphasic disease course was observed in TBE patients in China.^27, 57^

During the first large-scale TBE outbreak in China in 1952, the death rate and the proportion of patients with sequelae were both high (each was nearly one-third).^6^ Later, the case fatality rate of TBE dropped considerably (for example, 1.9% [23/1217] between 1995 and 2002 in Inner Mongolia).^27^ Timely diagnosis due to increased awareness of the disease and widely available laboratory tests and examinations, prompt patient transfer services as a result of improvements in the economy and means of transportation, and better treatment provided by supportive care following advances in medical sciences have contributed to the decrease in case fatality rate.^6^ However, sequelae of TBE are still common. Long-term disabilities were observed in nearly 20% (90/542) of TBE patients, with over one-third (32 cases) unable to work.^61^

No specific treatment for TBE exists, and management mainly involves symptom treatment and supportive care. Mongolian medicine treatment of TBE is also reported in the Inner Mongolia Region; however, these methods also involve supportive care as an adjuvant treatment.^27^

### Vaccines for TBE in China

The 1952 TBE outbreak in northeast China had a serious effect on the health of forest workers. As soon as TBE virus was isolated and characterized, vaccines against TBE were developed and manufactured. The first TBE vaccine became available in China in May 1953 to forest workers at high risk of TBE. This vaccine was a formalin-inactivated, whole-cell vaccine made of TBEV-infected mouse brain or chicken embryos.^6, 62^ Later, in 1960, a TBE vaccine derived from chicken embryo cell culture became widely available in China. In 1967, the first-generation TBE vaccine produced from primary hamster kidney (PHK) cell culture was manufactured and widely used. In 2004, a second-generation PHK vaccine (SenTaiBao, Changchun Institute of Biological Products Co., Ltd., Jilin, China) was approved in China for the prevention of TBE.^62^

TBEV strain Sen-Zhang (Far-Eastern subtype) is used in this second-generation purified PHK-inactivated vaccine. This vaccine is recommended for residents of TBEV-endemic areas and for people who enter endemic areas. The safety and immunogenicity of the vaccine have been established.^63, 64^ One study recruited healthy people aged 6–80 years who received 2 primary doses of the vaccine 14 days apart. The safety profile was acceptable after each dose. Anti-TBEV IgG antibody was detected by ELISA. At 4 weeks, 96% (486/506) had detectable antibodies with a geometric mean concentration (GMC) of 753 Vienna units per mL (VIEU/mL, range: 37–3190 VIEU/mL). At 6 months, 86% (434/502) remained IgG-positive with a GMC of 332 VIEU/mL (range: 42–3061 VIEU/mL), and at 1 year after the vaccination, 77% (386/502) were IgG-positive with a GMC of 225 VIEU/mL (range: 8–2225 VIEU/mL).^63^ In a second study, the immune response and the safety profile were investigated after a booster dose of the vaccine 18 months after the primary doses. The safety profile was acceptable. Among the 400 healthy participants (aged ≥8 years), GMC was 47 VIEU/mL (range: 0–124 VIEU/mL) before the booster dose. At 4 weeks after the booster dose, 96% (325/339) showed an immune response with a GMC of 3391 VIEU/mL. No difference in the percentage of subjects with an immune response was found among different age groups (that is, 8–16 years, 17–59 years, and ≥60 years), but GMC in the 8–16-year-old group was significantly higher (*P*<0.01). In addition,women were more likely to generate an immune response than men, and their GMC was significantly higher than that in men (*P*<0.05, respectively).^64^

Nevertheless, direct efficacy data for this second-generation, PHK-inactivated vaccine are lacking. Although it has been noted that among railway workers in Hailar, where TBE cases have been documented, no TBE cases have been reported in 2 consecutive years since the use of this second-generation PHK vaccine.^27^

## DISCUSSION

Located at the eastern end of the Eurasian continent, China, Japan and ROK all have reported prevalence of TBEV. All TBEV strains isolated from ROK are of the Western subtype, while those from China and Japan all belong to the Far-Eastern subtype, except for one TBEV strain obtained from the Altai Mountains in northwest China that has been most recently identified as the Siberian subtype. The main vectors of TBEV are *I. persulcatus* and *I. ovatus* in Japan; *H. longicornis*, *H. flava* and *I. nipponensis* in ROK; and *I. persulcatus*, *H. concinna*, *H. japonica*, *D. silvarum* and *I. ovatus* in China. In contrast to the common occurrence of TBEV in these three countries, the incidence of TBE cases varied greatly. In China, the first recorded TBE outbreak was in 1952, and TBE patients have since been diagnosed in the northeast, northwest, and southwest provinces and autonomous regions. TBE has been considered an occupational disease in China, and a national diagnosis standard has been issued by the Ministry of Health. To combat TBE, vaccines against TBEV have been manufactured and become available in China since 1953, and different vaccine types have been used during different periods over the past 60 years. Whereas in Japan, only four confirmed TBE patients have been recorded, and no TBE patient has yet been confirmed in ROK.

Various factors may have contributed to the differences observed in the prevalence of TBE in Japan, ROK and China. For instance, one study showed that the development of clinical TBE is the consequence of bites by ticks highly infected with TBEV.^65^ Although TBEV-infected ticks have been found in different regions of Japan and ROK, further investigation is needed to understand whether TBEV-infected ticks carry high numbers of viral RNA that are sufficient to cause TBE disease and what the density of such ticks is within a natural focus area. Because the dominant tick species are different in the TBEV-endemic areas in China, Japan and ROK, whether differences in host-seeking and feeding behavior among the ticks might play a role in the observed differences in TBE prevalence is worth analyzing.

Furthermore, differences in the virulence of TBEV isolates may also be the cause of the differences in the prevalence of TBE observed in Japan, ROK and China. Most recently, a TBEV strain (MucAr HB171/11) was isolated from *I. ricinus* in southeastern Germany, where only several human cases with mild gastrointestinal and constitutional symptoms, but no neurological symptoms, have been reported.^66^ Although further analysis is required to confirm the pathogenicity of this TBEV strain, the findings suggest that some TBE patients may only have constitutional symptoms. People infected with such low-virulence TBEV strains are not likely to show symptoms of encephalitis. These cases may be self-resolving (without seeking medical treatment) and therefore may fail to be recorded in TBE surveillance programs, which could result in a false low prevalence of TBEV infection. Animal experiments have already confirmed that the Japanese TBEV strain isolated from Hokkaido is less virulent in mice than other Far-Eastern subtype TBEV strains.^35^ One might also speculate that virulence is influenced by ticks transmitting the virus to humans. Further pathogenicity studies on the TBEV strains isolated from ROK, coupled with population-wide serological surveys to detect anti-TBEV antibodies for the confirmation of asymptomatic infections, may help us to understand the differences in TBE prevalence among these three East Asian countries.

Data from Russian and Lithuanian populations have demonstrated an association between human host genetics and the risk of TBE. Among Russian TBE patients, polymorphisms in various genes, including the OAS gene cluster (*OAS1*, *OAS2* and *OAS3*), are associated with a predisposition to the severe forms of TBE.^67^ In Lithuanian TBE patients, a 32-bp deletion in the chemokine receptor 5 (*CCR5*) gene was found to be a significant risk factor for clinical TBE regardless of age, while a polymorphism in the *TLR3* gene was associated with a predisposition to clinical TBE in adults but not in children (<18 years) and may also be associated with disease severity.^68, 69^ Furthermore, in different populations from North Eurasia, single nucleotide polymorphisms (SNPs) of the *OAS2* and *OAS3* genes have been found to be associated with predisposition to severe forms of TBE.^70^ Considering that disease susceptibility (for example, venous thromboembolism) has been known to vary between populations with different genetic backgrounds, such as those of Caucasian or East Asian descent, or even within East Asian populations (that is, the Chinese, the Japanese and the Koreans),^71^ insight into the differences in predisposition to TBEV and TBE prevalence in these East Asian countries may be provided if further studies are conducted to investigate the relationship between various genetic differences found among these populations.

JEV is also endemic to Japan, ROK and China. Effective vaccines against JEV became available in the 1950s. In Japan, a nationwide vaccination program against JEV started in 1954, which successfully provided protection to the public and significantly reduced the incidence of JEV infections.^72^ In ROK, an inactive vaccine derived from the mouse brain was first used between the late 1960s and the early 1970s, in 1985 the nationwide vaccination coverage for JEV reached ~90%, and the estimated rate of immunization was ~80% in 2008.^73^ Considering the presence of Flavivirus-induced antibody cross-reactivity,^74^ particularly that cross protection of JEV vaccines against other mosquito-borne flaviviruses, such as the West Nile virus,^75^ has been established and that people with pre-existing anti-JEV antibodies have shown low immune responses against TBEV,^42^ the populations in Japan and ROK (with their high vaccination rates for JEV) might have also been protected against TBEV because of an undiscovered cross-protection effect. Nevertheless, in 2013 among Japanese subjects aged 40–60 years, only <20% were reported to have anti-JEV antibodies,^72^ indicating a lack of potential cross protection of JEV vaccination against TBEV for the majority of this age group. In China, anti-JEV antibodies have been detected in the healthy population in Heilongjiang Province, where TBE is a notifiable disease,^76^ but whether anti-JEV antibody is prevalent in TBEV endemic regions is unclear. Vaccination against JEV started in China in 1968. In 1992, it was included in the Expanded Program on Immunization (EPI) in certain provinces and autonomous regions. In 2007, the JEV vaccination became one of the vaccines of the national EPI.^77^ Following nationwide vaccination against JEV in China, answering the question of whether a concomitant decrease in the incidence of TBE would occur requires long-term surveillance.

In addition, because the risk for TBEV infection in humans is relative to landscape, climate and host-species dynamics,^78^ differences in these variables among China, Japan and ROK may have contributed to the differences in TBE prevalence. Due to cultural, socioecological and educational differences in these countries, people’s behavior in preventing exposure to tick bites, such as avoiding entering tick habitats, using insect repellents that have confirmed efficacy against ticks and carrying out frequent checks for ticks and tick bites, can be expected to be different. For instance, as recently as 2013, there were few products available in Japan for repelling mites through skin application or clothing treatment; later that year, user instructions for repelling ticks were first added to some repellents, including those containing N,N-diethyl-m-toluamide (DEET).^79^ However, the extent to which the differences in preventive behavior have contributed to the differences in TBE prevalence in these three East Asian countries requires further investigation.

Nevertheless, in Japan and ROK, awareness of TBE is low, and not all clinicians are familiar with the symptoms of TBE and the relevant laboratory tests or examinations. Consequently, TBE patients might not be recognized and diagnosed correctly. By contrast, in China, the public and medical professionals in TBE-endemic areas have become aware of the disease since the early 1950s, and serological tests to assist the diagnosis of TBE have also been widely available. Patients with TBE are therefore more likely to be diagnosed correctly. To promote the awareness of TBE, ROK initiated a nationwide surveillance program in 2006 requesting all hospitals to submit samples from patients who had encephalitis with unknown causes to a central laboratory for TBEV-related tests. The program is ongoing, but as of 2016, no cases of TBE had been identified.

In the future, to obtain a comprehensive picture of TBE and the TBEV that is endemic to China, Japan and ROK, further investigations are required. Biological characteristics (such as feeding habits) of various dominant tick species in each TBEV-endemic area within different countries may need to be compared in order to identify the risk level of acquiring TBE in these areas. The virulence and pathogenesis of the TBEV strains obtained from different countries, as well as population genetics for disease susceptibility, should be compared in order to answer key questions, such as whether patients infected with TBEV in Japan and ROK are mostly asymptomatic and rarely show any encephalitis symptoms. Moreover, public health education programs may need to be developed to increase awareness of tick-borne diseases, particularly TBE (including knowledge of disease transmission risk from tick bites, protection measures and symptom recognition). For medical professionals, exposure to TBE-relevant information may need to be increased systematically.

Large-scale serological surveillance to identify anti-TBEV antibodies in areas where TBEV has been isolated should be carried out both in humans and in domestic and wild animal hosts to aid the understanding of disease prevalence. To this end, detection methods with high sensitivity and high specificity for TBEV that can be performed in more laboratories, such as diagnostic ELISA using subviral particles,^40, 41^ are available. In TBEV-endemic areas, vigilant monitoring of climate and landscape changes and the dynamics of vector species, as well as the wild and domestic animal hosts involved in the TBEV lifecycle, should be established to provide information for forecasting TBE risk for humans. As demonstrated by the European experience, vaccinations against TBEV infection can significantly decrease the incidence of TBE.^1, 2^ Considering that vaccines against TBE have been used in China for over 60 years and have shown satisfactory immunogenicity,^27^ and that vaccines developed in Japan have also shown protective effects in mice,^80^ it is feasible to design an effective vaccination plan for specified TBEV-endemic areas where the risk of TBE has been identified, providing it is supported by cost-effectiveness analysis.

In conclusion, a potential medical burden caused by TBEV exists in Japan and ROK, while in China, the challenge is to control endemic TBEV as effectively as possible via public health educational programs and population-wide vaccination. Therefore, establishing a surveillance system targeting TBEV and TBE disease, perhaps through the collaboration of these countries, may be of great benefit to public health, as well as to the health of domestic animals and wildlife.

## Figures and Tables

**Table 1:** Tick species infected with tick-borne encephalitis virus in regions of Japan, Republic of Korea and China

**Country**	**Tick species**	**Sampling region**
Japan^11^	*Ixodes ovatus*	Southern part of Hokkaido
		
The Republic of Korea	*I. nipponensis*	Yangpyeong and Dongducheon, Gyeonggi-do; Pyeongchang and Jeongseon, Gangwon-do
2005–2006 study^14, 15^	*Haemaphysalis flava*	Yangpyeong, Gyeonggi-do; Pyeongchang, Gangwon-do
	*H. japonica*	Jeongseon, Gangwon-do
	*H. longicornis*	Dongducheon, Gyeonggi-do; Pyeongchang and Jeongseon, Gangwon-do
		
2007 study^16^	*H. flava*	Jeju Island
	*H. longicornis*	Jeju Island
		
2011–2012 study^17^	*I. niponensis, H. flava, and H. longicornis*	Chuncheon and Sokcho, Gangwon-do; Gunsan, Jeollabuk-do; Jeju Island
		
2014 study^18^	*I. nipponensis*	Andong, Gyeongsangbuk-do
	*H. flava*	Andong and Uiseong, Gyeongsangbuk-do; Dong-gu, Daegu metropolitan area
	*H. longicornis*	Uiseong, Gyeongsangbuk-do; Yangsan, Gyeongsangnam-do
		
China^5, 6, 20, 23, 24, 25, 26, 27, 28^	*I. persulcatus*, *H. concinna*, *H. japonica*, and *Dermacentor silvarum*	Changbaishan, Daxing'anling, and Xiaoxing'anling in Heilongjiang, Jilin, and Inner Mongolia Autonomous Region in the northeast; Tianshan and Altay Mountains in the Xinjiang Uygur Autonomous Region in the northwest
	*I. ovatus*	Hengduan Mountain Range and Gaoligongshan in Yunnan in the southwest

## References

[bib1] Barrett PN, Portsmouth D, Ehrlich HJ Tick-borne encephalitis virus vaccines. In: Plotkin SA, Orenstein WA, Offit PA (eds). Vaccines Sixth Edition, Philadelphia: Elsevier Saunders. 2013, 773–788.

[bib2] Suss J. Tick-borne encephalitis in Europe and beyond—the epidemiological situation as of 2007. Euro Surveill 2008; 13: pii: 18916. 18761916

[bib3] Mansfield KL, Johnson N, Phipps LP et al. Tick-borne encephalitis virus—a review of an emerging zoonosis. J Gen Virol 2009; 90: 1781–1794. 19420159 10.1099/vir.0.011437-0

[bib4] Waldenstrom J, Lundkvist A, Falk KI et al. Migrating birds and tickborne encephalitis virus. Emerg Infect Dis 2007; 13: 1215–1218. 17953095 10.3201/eid1308.061416 PMC2828075

[bib5] Liu YJ, Jiang YT, Guo CS et al. Epidemiological characteristics of tick borne encephalitis natural foci in Jilin. Zhongguo Ren Min Jie Fang Jun Jun Shi Yi Xue Ke Xue Yuan Yuan Kan (Bull Acad Military Med Sci) 1979; 3: 109–120 Chinese.

[bib6] Yin DM, Liu RZ. Review on the control of forest encephalitis in the forest areas of north-east China. Zhonghua Liu Xing Bing Xue Za Zhi (Chin J Epidemiol) 2000; 21: 387–389 Chinese.

[bib7] Okuno T, Oya A, Ito T. The identification of Negishi virus, a presumably new member of Russian spring-summer encephalitis virus family isolated in Japan. Jpn J Med Sci Biol 1961; 14: 51–59. 14481659 10.7883/yoken1952.14.51

[bib8] Venugopal K, Buckley A, Reid HW et al. Nucleotide sequence of the envelope glycoprotein of Negishi virus shows very close homology to louping ill virus. Virology 1992; 190: 515–521. 1326816 10.1016/0042-6822(92)91245-p

[bib9] Morita K, Igarashi A, Sato T et al. A suspected case of tick-borne encephalitis in Hokkaido. Infect Agents Surveill Rep 1994; 15: 273–274 (Japanese).

[bib10] Takashima I, Morita K, Chiba M et al. A case of tick-borne encephalitis in Japan and isolation of the the virus. J Clin Microbiol 1997; 35: 1943–1947. 9230360 10.1128/jcm.35.8.1943-1947.1997 PMC229881

[bib11] Takeda T, Ito T, Chiba M et al. Isolation of tick-borne encephalitis virus from *Ixodes ovatus* (Acari: Ixodidae) in Japan. J Med Entomol 1998; 35: 227–231. 9615539 10.1093/jmedent/35.3.227

[bib12] Ministry of Health. Labour and Welfare/National Institute of Infectious Diseases, Japan. Infect Dis Wkly Rep Jpn 2016; 18 (week 32) 3 (Japanese).

[bib13] Zheng YC, Yang XK, Zhang LJ. Demographic characteristics of patients with forest encephalitis. Zhongguo Ren Shou Gong Huan Bing Za Zhi (Chin J Zoonoses) 2003; 19: 18 (Chinese).

[bib14] Kim S-Y, Yun S-M, Han MG et al. Isolation of tick-borne encephalitis viruses from wild rodents, South Korea. Vector Borne Zoonotic Dis 2008; 8: 7–13. 18240970 10.1089/vbz.2006.0634

[bib15] Kim SY, Jeong YE, Yun SM et al. Molecular evidence for tick-borne encephalitis virus in ticks in South Korea. Med Vet Entomol 2009; 23: 15–20. 19239610 10.1111/j.1365-2915.2008.00755.x

[bib16] Ko S, Kang J-G, Kim SY et al. Prevalence of tick-borne encephalitis virus in ticks from southern Korea. J Vet Sci 2010; 11: 197. 20706026 10.4142/jvs.2010.11.3.197 PMC2924480

[bib17] Yun S-M, Song BG, Choi W et al. Prevalence of tick-borne encephalitis virus in ixodid ticks collected from the Republic of Korea during 2011–2012. Osong Public Health Res Perspect 2012; 3: 213–221. 24159517 10.1016/j.phrp.2012.10.004 PMC3747658

[bib18] Yun S-M, Lee Y-J, Choi W et al. Molecular detection of severe fever with thrombocytopenia syndrome and tick-borne encephalitis viruses in ixodid ticks collected from vegetation, Republic of Korea, 2014. Ticks Tick Borne Dis 2016; 7: 970–978. 27211914 10.1016/j.ttbdis.2016.05.003

[bib19] Donoso Mantke O, Schadler R, Niedrig M. A survey on cases of tick-borne encephalitis in European countries. Euro Surveill 2008; 13: pii: 18848. 18445446

[bib20] Lu Z, Bröker M, Liang G. Tick-borne encephalitis in mainland China. Vector Borne Zoonotic Dis 2008; 8: 713–720. 18837668 10.1089/vbz.2008.0028

[bib21] Kentaro Y, Yamazaki S, Mottate K et al. Genetic and biological characterization of tick-borne encephalitis virus isolated from wild rodents in southern Hokkaido, Japan in 2008. Vector Borne Zoonotic Dis 2013; 13: 406–414. 23590320 10.1089/vbz.2012.1231 PMC3669602

[bib22] Hayasaka D, Suzuki Y, Kariwa H et al. Phylogenetic and virulence analysis of tick-borne encephalitis viruses from Japan and far-eastern Russia. J Gen Virol 1999; 80 (Pt 12) 3127–3135. 10567643 10.1099/0022-1317-80-12-3127

[bib23] Bi WM, Deng HP, Bu XY. Regionalisation of natural foci of forest encephalitis. Shou Du Shi Fan Da Xue Xue Bao (J Cap Normal Univ: Natl Sci Ed) 1997; 18: 100–107 (Chinese).

[bib24] Xie XC, Yu X, Zhang TX et al. A survey report on the natural foci of Russian spring-summer encephalitis in the mountainous areas of Tianshan and Altay mountains in Xinjiang. Di Fang Bing Tong Bao (Endemic Dis Bull) 1991; 6: 109–114 Chinese.

[bib25] Zhang GL, Liu R, Sun X et al. Investigation on the endemic foci of tick-borne encephalitis virus in Xiaerxili Natural Reserve, Xinjiang. Zhonghua Liu Xing Bing Xue Za Zhi (Chin J Epidemiol) 2013; 34: 438–442 (Chinese). 24016430

[bib26] Huang WL, Hou ZL, Zi DY et al. Investigation of the Russian spring-summer encephalitis virus in Yunnan province. Zhongguo Yu Fang Shou Yi Xue Bao (Chin J Prev Veterinary Med) 2001; 23: 231–233 (Chinese).

[bib27] Xing Y, Schmitt HJ, Arguedas A et al. Tick-borne encephalitis in China: A review of epidemiology and vaccines. Vaccine 2017; 35: 1227–1237. 28153343 10.1016/j.vaccine.2017.01.015

[bib28] Sun X, Zhang GL, Liu R et al. Endemic foci of tick-borne encephalitis in Altai Mountains in Xinjiang. Zhongguo Ren Shou Gong Huan Bing Xue Bao (Chin J Zoonoses) 2015; 31: 1189–1192 (Chinese).

[bib29] Miyamoto K, Nakao M, Uchikawa K et al. Prevalence of Lyme borreliosis spirochetes in ixodid ticks of Japan, with special reference to a new potential vector, *Ixodes ovatus* (Acari: Ixodidae). J Med Entomol 1992; 29: 216–220. 1495032 10.1093/jmedent/29.2.216

[bib30] Grešíková M Tickborne encephalitis. In: Hui YH, Gorham JR, Murrell KD, Cliver DO (eds).Foodborne Disease Handbook. Vol. 2. Diseases Caused by Viruses, Parasites, and Fungi. New York: Marcel Dekker. 1994, 113–135.

[bib31] Takeda T, Ito T, Osada M et al. Isolation of tick-borne encephalitis virus from wild rodents and a seroepizootiologic survey in Hokkaido, Japan. Am J Trop Med Hyg 1999; 60: 287–291. 10072153 10.4269/ajtmh.1999.60.287

[bib32] Yoshii K, Mottate K, Omori-Urabe Y et al. Epizootiological study of tick-borne encephalitis virus infection in Japan. J Vet Med Sci 2011; 73: 409–412. 21060247 10.1292/jvms.10-0350

[bib33] Miyamoto K, Nakao M, Fujita H et al. The ixodid ticks on migratory birds in Japan and isolation of Lyme disease spirochetes from bird-feeding ticks. Jpn J Sanit Zool 1993; 44: 315–326.

[bib34] Goto A, Hayasaka D, Yoshii K et al. Genetic and biological comparison of tick-borne encephalitis viruses from Hokkaido and far-eastern Russia. Jpn J Vet Res 2002; 49: 297–307. 11949477

[bib35] Chiba N, Iwasaki T, Mizutani T et al. Pathogenicity of tick-borne encephalitis virus isolated in Hokkaido, Japan in mouse model. Vaccine 1999; 17: 779–787. 10067683 10.1016/s0264-410x(98)00262-x

[bib36] Yoshii K, Moritoh K, Nagata N et al. Susceptibility to flavivirus-specific antiviral response of Oas1b affects the neurovirulence of the Far-Eastern subtype of tick-borne encephalitis virus. Arch Virol 2013; 158: 1039–1046. 23266832 10.1007/s00705-012-1579-1

[bib37] Sakai M, Yoshii K, Sunden Y et al. Variable region of the 3' UTR is a critical virulence factor in the Far-Eastern subtype of tick-borne encephalitis virus in a mouse model. J Gen Virol 2014; 95: 823–835. 24394696 10.1099/vir.0.060046-0

[bib38] Mandl CW, Holzmann H, Meixner T et al. Spontaneous and engineered deletions in the 3' noncoding region of tick-borne encephalitis virus: construction of highly attenuated mutants of a flavivirus. J Virol 1998; 72: 2132–2140. 9499069 10.1128/jvi.72.3.2132-2140.1998 PMC109508

[bib39] Formanová P, Černý J, Bolfíková BČ et al. Full genome sequences and molecular characterization of tick-borne encephalitis virus strains isolated from human patients. Ticks Tick Borne Dis 2015; 6: 38–46. 25311899 10.1016/j.ttbdis.2014.09.002

[bib40] Obara M, Yoshii K, Kawata T et al. Development of an enzyme-linked immunosorbent assay for serological diagnosis of tick-borne encephalitis using subviral particles. J Virol Methods 2006; 134: 55–60. 16540179 10.1016/j.jviromet.2005.11.018

[bib41] Inagaki E, Sakai M, Hirano M et al. Development of a serodiagnostic multi-species ELISA against tick-borne encephalitis virus using subviral particles. Ticks Tick Borne Dis 2016; 7: 723–729. 26969490 10.1016/j.ttbdis.2016.03.002

[bib42] Chiba N, Osada M, Komoro K et al. Protection against tick-borne encephalitis virus isolated in Japan by active and passive immunization. Vaccine 1999; 17: 1532–1539. 10195790 10.1016/s0264-410x(98)00360-0

[bib43] Kim CM, Yi YH, Yu DH et al. Tick-borne rickettsial pathogens in ticks and small mammals in Korea. Appl Environ Microbiol 2006; 72: 5766–5776. 16957192 10.1128/AEM.00431-06 PMC1563606

[bib44] Kang JG, Kim HC, Choi CY et al. Molecular detection of *Anaplasma**Bartonella*, and *Borrelia* species in ticks collected from migratory birds from Hong-do Island, Republic of Korea. Vector Borne Zoonotic Dis 2013; 13: 215–225. 23428091 10.1089/vbz.2012.1149

[bib45] Kaiser R. Tick-borne encephalitis. Infect Dis Clin North Am 2008; 22: 561–575. 18755391 10.1016/j.idc.2008.03.013

[bib46] Yu X-J, Liang M-F, Zhang S-Y et al. Fever with thrombocytopenia associated with a novel bunyavirus in China. N Engl J Med 2011; 364: 1523–1532. 21410387 10.1056/NEJMoa1010095 PMC3113718

[bib47] Kato H, Yamagishi T, Shimada T et al. Epidemiological and clinical features of severe fever with thrombocytopenia syndrome in Japan, 2013-2014. PLoS One 2016; 11: e0165207. 27776187 10.1371/journal.pone.0165207 PMC5077122

[bib48] Song ZM, Han L, Hong CL et al. Studies on epidemic foci of tick-borne encephalitis (TBE) surveillance and TBE virus isolated. Sheng Wu Ji Shu Tong Xue (Lett Biotechnol) 2004; 15: 43–44.

[bib49] Xu SQ. Population ecology of Ixodes persulcatus and tick-borne pathogens, PhD thesis Shijiazhuang, China: Hebei Normal University. 2015. Retrieved from: WanFang Data (http://d.wanfangdata.com.cn/Thesis/Y2788690) (Chinese).

[bib50] Liu R, Zhang G, Liu X et al. Detection of the Siberian tick-borne encephalitis virus in the Xinjiang Uygur Autonomous Region, northwestern China. Bing Du Xue Bao (Chin J Virology) 2016; 32: 26–31 Chinese. 27295880

[bib51] Lundkvist K, Vene S, Golovljova I et al. Characterization of tick-borne encephalitis virus from Latvia: evidence for co-circulation of three distinct subtypes. J Med Virol 2001; 65: 730–735. 11745938 10.1002/jmv.2097

[bib52] Zhang DH, Zhang ZX, Wang YM et al. Trends of forest encephalitis endemic in Heilongjiang. Ji Bing Jian Ce (Dis Surveill) 2000; 15: 57–58 Chinese.

[bib53] Li H, Zhang XG. Epidemic situation of forest encephalitis in Da Xing An Ling forest areas in Inner Mongolia. Zhonghua Yu Fang Yi Xue Za Zhi (Chin J Prev Med) 1999; 33: 148 Chinese.

[bib54] Yan DC, Li YG, Wang SY et al. Tick-borne encephalitis virus was isolated from the forest areas of Da Xing An Ling in Inner Mongolia for the first time. Neimenggu Yi Xue Za Zhi (Inner Mongolia Med J) 1996; 16: 65–67 Chinese.

[bib55] Zhang HL, Zhang YZ, Yang WH et al. Serological survey of anti-viral antibodies in human and other animals in downstream Lancang River, Yunnan Province, China. Yi Xue Dong Wu Fang Zhi (Chin J Pest Control) 2004; 20: 207–211 Chinese.

[bib56] National Health and Family Planning Commission of the People's Republic of ChinaICS 13.100 C60—Diagnostic criteria of occupational forest encephalitis (GBZ 88–2002). Beijing: NHFPC, China. 2002. Available at: http://www.moh.gov.cn/cmsresources/zwgkzt/wsbz/zybzdbz/zyb/zyb/088.pdf (accessed 20 June 2017) (Chinese.

[bib57] Mao LJ, Zhao JY, Xu XX et al. Clinical analysis on 380 cases of forest encephalitis. Zhongguo Gong Ye Yi Xue Za Zhi (Chin J Ind Med) 2002; 15: 137–140 Chinese.

[bib58] Zhang XG, Cui W, Tong YQ. Clinical analysis of 30 tick-born encephalitis patients with eye disease. Neimenggu Yi Xue Za Zhi (Inner Mongolia Med J) 2004; 36: 581–582 Chinese.

[bib59] Wang GJ, Zhang XG, Sun X. Clinical analysis of tick-borne encephalitis patients with cardiac damage. Neimenggu Yi Xue Za Zhi (Inner Mongolia Med J) 2012; 44: 176–179.

[bib60] Chen Y, Zhang XG, Han SZ. Clinical observation of 93 patients with tick-borne encephalitis complicated with liver function impairment. Zhongguo Xian Dai Yao Wu Ying Yong (Chin J Mod Drug Appl) 2012; 6: 51–52 Chinese.

[bib61] Li H. Analysis of 90 cases of disability as the sequela of forest encephalitis. Zhongguo Lin Chuang Kang Fu (Chin J Clin Rehabil) 2002; 6: 374 Chinese.

[bib62] Song ZM, Liu YB, Huang YC. Research advances in vaccines of tick-borne encephalitis. Wei Sheng Wu Xue Mian Yi Xue Jin Zhan (Prog Microbiol Immunol) 2005; 33: 56–59 Chinese.

[bib63] He SY, Fu B, Fan XB et al. Immunogenicity and safety of a tick-borne encephalitis inactivated vaccine. Zhongguo Shi Yan Zhen Duan Xue (Chin J Lab Diag) 2014; 18: 810–811 Chinese.

[bib64] He SY, Wang YW, Qiao JG et al. Clinical trial of booster immune effect of inactivated tick-borne encephalitis vaccine. Zhongguo Sheng Wu Zhi Pin Xue Za Zhi (Chin J Biol) 2015; 28: 377–378.

[bib65] Kovalevskii IUV, Korenberg EI. Factors that determine the possibility of tick-borne encephalitis infection. 3. The probability of human contact with an infected vector in the central taiga forests of Khabarovsk Territory. Med Parazitol (Mosk) 1990; 3: 5–8 Russian. 2215376

[bib66] Dobler G, Bestehorn M, Antwerpen M et al. Complete genome sequence of a low-virulence tick-borne encephalitis virus strain. Genome Announc 2016; 4: e01145–e01116. 27795275 10.1128/genomeA.01145-16 PMC5073262

[bib67] Barkhash AV, Perelygin AA, Babenko VN et al. Variability in the 2'-5'-oligoadenylate synthetase gene cluster is associated with human predisposition to tick-borne encephalitis virus-induced disease. J Infect Dis 2010; 202: 1813–1818. 21050126 10.1086/657418

[bib68] Kindberg E, Mickiene A, Ax C et al. A deletion in the chemokine receptor 5 (CCR5) gene is associated with tickborne encephalitis. J Infect Dis 2008; 197: 266–269. 18179389 10.1086/524709

[bib69] Mickiene A, Pakalniene J, Nordgren J et al. Polymorphisms in chemokine receptor 5 and Toll-like receptor 3 genes are risk factors for clinical tick-borne encephalitis in the Lithuanian population. PLoS One 2014; 9: e106798. 25226020 10.1371/journal.pone.0106798 PMC4165893

[bib70] Barkhash AV, Babenko VN, Kobzev VF et al. Polymorphism in the human 2'-5'-oligoadenylate synthetase genes (*OAS*, associated with predisposition to severe forms of tick-borne encephalitis, in populations from North Eurasia. Mol Biol (Mosk) 2010; 44: 985–993 (Russian). 21290821

[bib71] Miyata T, Maruyama K, Banno F et al. Thrombophilia in East Asian countries: are there any genetic differences in these countries? Thromb J 2016; 14 (Suppl 1): 25. 27766051 10.1186/s12959-016-0109-x PMC5056495

[bib72] Itoh K, Iwamoto K, Satoh Y et al. Knowledge obtained from an elderly case of Japanese encephalitis. Intern Med 2016; 55: 2487–2490. 27580555 10.2169/internalmedicine.55.6646

[bib73] Lee DW, Choe YJ, Kim JH et al. Epidemiology of Japanese encephalitis in South Korea, 2007–2010. Int J Infect Dis 2012; 16: e448–e452. 22497964 10.1016/j.ijid.2012.02.006

[bib74] Mansfield KL, Horton DL, Johnson N et al. Flavivirus-induced antibody cross-reactivity. J Gen Virol 2011; 92 (Pt 12): 2821–2829. 21900425 10.1099/vir.0.031641-0 PMC3352572

[bib75] Lobigs M, Diamond MS. Feasibility of cross-protective vaccination against flaviviruses of the Japanese encephalitis serocomplex. Expert Rev Vaccines 2012; 11: 177–187. 22309667 10.1586/erv.11.180 PMC3337329

[bib76] An ZJ, Ma YJ, Bo F et al. Analysis on surveillance data regarding the level of Japanese encephalitis antibody in Heilongjiang province, 2006. Zhonghua Liu Xing Bin Xue Za Zhi (Chin J Epidemiol) 2007; 28: 1186–1189 (Chinese). 18476578

[bib77] Gao X, Li X, Li M et al. Vaccine strategies for the control and prevention of Japanese encephalitis in Mainland China, 1951–2011. PLoS Negl Trop Dis 2014; 8: e3015. 25121596 10.1371/journal.pntd.0003015 PMC4133196

[bib78] Kiffner C, Zucchini W, Schomaker P et al. Determinants of tick-borne encephalitis in counties of southern Germany, 2001–2008. Int J Health Geogr 2010; 9: 42. 20707897 10.1186/1476-072X-9-42 PMC2928184

[bib79] Ogawa K, Komagata O, Hayashi T et al. Field and laboratory evaluations of the efficacy of DEET repellent against ixodes ticks. Jpn J Infect Dis 2016; 69: 131–134. 26073735 10.7883/yoken.JJID.2015.038

[bib80] Omori-Urabe Y, Yoshii K, Ikawa-Yoshida A et al. Needle-free jet injection of DNA and protein vaccine of the Far-Eastern subtype of tick-borne encephalitis virus induces protective immunity in mice. Microbiol Immunol 2011; 55: 893–897. 22004565 10.1111/j.1348-0421.2011.00389.x

